# MAP17 (PDZKIP1) as a novel prognostic biomarker for laryngeal cancer

**DOI:** 10.18632/oncotarget.3470

**Published:** 2015-02-28

**Authors:** María-José de Miguel-Luken, Manuel Chaves-Conde, Verónica de Miguel-Luken, Sandra Muñoz-Galván, José Luis López-Guerra, Juan C. Mateos, Jerónimo Pachón, David Chinchón, Vladimir Suarez, Amancio Carnero

**Affiliations:** ^1^ Department of Medical Oncology, Hospital Universitario Virgen del Rocío, Seville, Spain; ^2^ University of Málaga, Málaga, Spain; ^3^ Instituto de Biomedicina de Sevilla, IBIS, Hospital Universitario Virgen del Rocío, Universidad de Sevilla, Consejo Superior de Investigaciones Científicas, Seville, Spain; ^4^ Department of Radiation Oncology, Hospital Universitario Virgen del Rocío, Seville, Spain; ^5^ Department of Pathology, Hospital Universitario Virgen del Rocío, Seville, Spain

**Keywords:** MAP17, ROS, SGLT, biomarker, larynx/laryngeal cancer

## Abstract

Larynx cancer organ preservation treatments with chemo and radiotherapy have substantially improved laryngoesophageal dysfunction-free survival. However, both of them lead to a high incidence of acute and chronic toxicities and a significant number of patients relapse. To date, there is no evidence available to establish the group of patients that may benefit from preservation approaches and clinical criteria such as primary tumor extension or pretreatment tracheotomy are not validated. MAP17 is a small non-glycosylated membrane protein overexpressed in carcinomas. The tumoral behavior induced by MAP17 is associated with reactive oxygen species production in which SGLT1 seems involved. In this study we found that the levels of MAP17 were related to clinical findings and survival in a cohort of 58 patients with larynx cancer. MAP17 expression is associated with overall survival (p<0.001) and laryngoesophageal dysfunction-free survival (p=0.002). Locoregional control in patients with high MAP17 showed better outcomes than those with low MAP17 (p=0.016). Besides, a positive correlation was observed between MAP17 expression and SGLT (p=0.022) and the combination of high levels of MAP17/SGLT also led to an increased overall survival (p=0,028). These findings suggest that MAP17, alone or in combination with SGLT1, may become a novel predictive biomarker for laryngeal carcinoma.

## INTRODUCTION

Squamous cell carcinoma of the head and neck represents 4% of all cancers diagnosed worldwide, with >500.000 new cases recorded in 2008. Of them, 151.000 cases (130.000 men and 21.000 women) were laryngeal cancer with an estimated age-standardized world mortality rate of 2.3/100.000 habitants. In fact, 82.000 deaths were estimated in 2008 due to this cause [1].

Alcohol and tobacco abuse are common etiologic factors [2] but exposure to hard-alloys dust, chlorinated solvents [3] and familiar genetic patterns [4] have been also implicated. The role of Human Papilomavirus (HPV) is well established for squamous cell carcinoma of the oropharynx but it remains unclear for laryngeal cancer [5]. Besides, these patients are at risk of developing second primary tumors due to chronic aerodigestive tract carcinogen exposure: 14% in 5 years, 26% in 10 years and 37% in 15 years [6].

The main prognostic factor for overall survival (OS) is tumor staging, where node invasion is more relevant than tumor extension [7]. Other OS prognostic factors are patient's comorbidity, performance status-ECOG (PS) [8], persistent toxic consumption habits [9], second primary tumor appearance [10] and primary tumor localization. In particular, glottic tumors reach 81% OS rates meanwhile supraglottic tumors drop to 70%, probably due to early detection [11]. Moreover, PS [8,12], node invasion [12, 13] and localization [8] are prognostic factors for disease-free survival in conjunction with pathologic stage (pT) [13], surgical resection margins [12] and pretreatment tracheotomy [14].

Until the 1980s, total laryngectomy surgery was the standard treatment with subsequent loss of speech and airway patency [15]. Consequently, treatment aims changed in order to improve patient's quality of life through larynx sparing approaches. Organ preservation treatments for laryngeal cancer patients depend on whether the tumor is presented in early stages (I and II) or advanced locoregional disease (stage III/IV). In general, early stages are treated with either primary surgery or definitive radiotherapy (RT). Five-year OS in patients with stage I or stage II disease is typically 70 to 90%. Surgery and RT seem to have similar effectiveness in this setting although they have not been compared in a randomized trial [16, 17]. Advanced disease requires multimodal approach, usually a combination of chemotherapy (CT) or biotherapy (B) with cetuximab plus RT. Although functional organ sparing approaches permit larynx preservation they do not provide a survival advantage over total laryngectomy [18]. Currently, three sparing approaches are accepted: RT, bio/chemo/radiotherapy (B/CTRT) and induction CT (ICT) followed by B/CTRT.

Regarding B/CTRT, cisplatin showed higher preservation rates as compared with induction CT followed by RT or RT alone (88% vs. 75% and 70%, respectively) with similar two and five year survival [19]. Later, a 10-year follow up publication confirmed that the arms that included CT improved laryngectomy-free survival [20]. Besides, a subsequent meta-analysis for locally advanced larynx cancer found that adding CT concomitant with RT led to a benefit of 6.5% absolute improvement in 5-year OS [21]. As for bio-radiotherapy, RT plus cetuximab showed better locoregional control rates than RT alone for advanced head and neck cancers [22]. A subset analysis of this study with hypopharyngeal and laryngeal carcinoma patients showed a hazard ratio (HR) 0.62 preservation in the cetuximab arm but this was not statistically significant [23].

With respect to ICT, the Veterans study demonstrated 64% preservation larynx rate without worsening survival in the induction followed by RT arm compared with surgery plus RT [24]. Afterwards, induction treatments were developed and the docetaxel, cisplatin and 5-Fluorouracil (DCF) schedule became the standard treatment, preserving the larynx 15% more than cisplatin and 5-Fluorouracil [25]. Furthermore, cetuximab has been studied as a concurrent treatment with RT instead of cisplatin after DCF induction chemotherapy, showing similar larynx preservation results in a phase II trial [26].

However, these strategies of treatment entail up to a 43% rate of late toxicities [27] and have not shown to prolong OS more than radical treatments. Interestingly, 5-year OS has decreased from 67.4% in 1985 to 59.6% in 2006 despite the development of new therapies for larynx cancer (Source: Surveillance, Epidemiology and End Results Program. Accessed: http://seer.cancer.gov/). One of the reasons that could justify this issue is that preservation approaches were not broadly used until the time of database collection. These two facts lead to the need for developing predictive biomarkers in order to select the patients that may benefit from preservation techniques and are not going to suffer unnecessary toxicities.

MAP17 is a small 17 Kda membrane protein present in a high proportion of tumors, not only in carcinoma. It has been found present in adenoma and benign tumors, and is highly expressed in metastatic carcinoma. Furthermore, its expression correlates with staging and malignant status of the tumor [28]. The expression is mainly driven at a transcriptional level either by promoter activation or demethylation [29, 30]. Expression of MAP17 in primary cells triggers senescence through p38, but in tumoral cells enhances the malignant capabilities of these cells, increasing proliferation, migration, resistance to apoptosis, etc [31, 32]. MAP17 expression increases the levels of reactive oxygen species (ROS) in cells which may account for some of the increased tumoral properties [33]. In turn, a further increase of ROS might switch the balance towards apoptosis. Thus, MAP17 may increase the efficacy of therapies increasing ROS and therefore constitute a biomarker for better prognosis of these tumors. In cervix tumors treated with cisplatin and radiotherapy, high levels of MAP17 mark good survival of the patients [30]. Therefore, MAP17 is not only a marker for stage and malignant status but also may be a marker of prognosis and response to therapies involving oxidative stress. In this manuscript we have explored the relevance of the presence of MAP17 in larynx tumors where primary response is mainly achieved by treatments with radiotherapy and cisplatin or other radiosensitizers.

## RESULTS

### Clinical cohort description

At the time of the analysis, 21 (32%) deaths and 31 (48%) recurrences had occurred with a median follow-up of 29 months. The most common cause of LDS failure was local recurrence, which required salvage laryngectomy in 16 (52%) of cases. Mean OS was 58.2 months (48.7-67.7, CI 95%) and mean LDS 44.6 (35.2-54.1, CI 95%). Lymph node metastases were significantly associated with decreased OS (N0: 63.1 m, N1: 38.6 and N2 22.2 m, p=0.019) while tumor local extension impacted LDS negatively (T4 extension 7.3 m vs. 47.1m non T4 extension, p=0.003). Besides, patients who required pretreatment tracheotomy had significantly worse LDS (54.3 vs. 18.9 months, p=0.001). The two-year cumulative proportion of patients with larynx preservation and OS were 57% and 76% respectively. Besides, locoregional control rate at two years was 60%. Therefore, our cohort behaves similarly to others reported in the literature [19,24].

### MAP17 expression in larynx tumor samples

**Figure 1:**
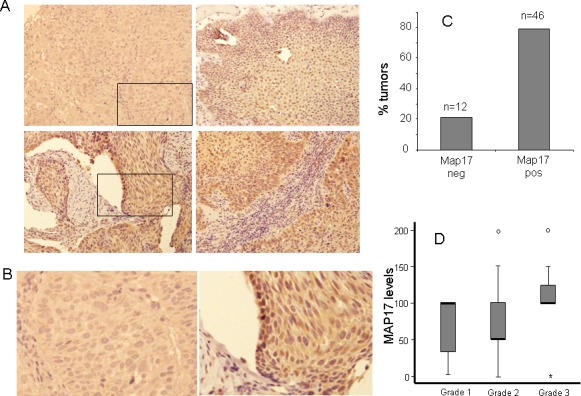
MAP17 overexpression in larynx tumors A) Representative images of MAP17 immunostaining are shown for different larynx tumors. B) High magnification of M17 positive and negative tumors. The picture shows a magnification of the inset of figure A. C) A graph is shown representing the percentage of laryngeal tumors with dichotomous MAP17 levels. The score for positive tumors were >62. D) The distribution of the MAP17 expression levels among different grades of larynx tumors is shown. The MAP17 levels (score) refers to maximum levels (0–2) scored by the percentage of cells (0–100). The normalized levels were obtained by multiplying the percentage of cells by the level of intensity observed. Anova test was performed to establish the statistical association between MAP17 protein levels and the grade of the tumor (p<0,05).

**Figure 2:**
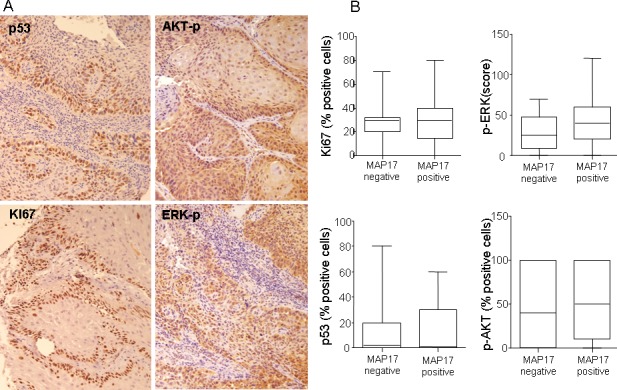
p53, Ki67, p-ERK or p-AKT do not show correlation with MAP17 expression in larynx tumors A) Representative images of p53, Ki67, p-ERK or p-AKT immunostainings are shown for larynx tumors. B) Graphs showing lack of correlation between these proliferative or antiapoptotic markers and MAP17 expression.

Out of 65 samples, only 58 were analyzed for MAP17 expression, either due to technical problems or because they did not contain any tumor cellularity. Out of the 58 samples, 46 (79%) were positives for MAP17 expression (Figure 1A, B and C) and there was a trend showing higher levels of MAP17 in advanced grades of the tumor (Figure 1D), although in this case, probably due to the low number of cases, it was not statistically significant. Surrounding normal tissue did not express MAP17 or expressed very low levels. We also analyzed other markers for proliferation such as KI67 or the activated form of ERK (phosphorylated ERK, ERK-p), or apoptosis such as mutant p53 or activated AKT (phosphorylated AKT, AKT-p). Our cohort showed a percentage of samples positive for KI67, mutant p53, ERK-p or AKT-p, but these groups did not show correlation with MAP17 levels (Figure 2). However, KI67 positivity showed statistically significant correlation with OS (Table 2). No correlation of MAP17 was observed with clinical parameters such as tumor localization, smoking habit, alcohol consumption, tumoral stage, pre-treatment tracheotomy and development of acute toxicities during chemoradiotherapy.

**Table 1:** Population characteristics and treatment

Characteristics	No. %	
Mean age	62 years	
Male	61	94
Squamous cell carcinoma	65	100
Cigarette smoking		
Current smokers	44	68
Former smokers	19	29
Never smokers	2	3
Smokers of ≥ 10 pack-years	60	92
Regular alcohol intake	46	71
PS 0-1	62	95
Pretreatment tracheotomy	21	32
Localization		
Supraglottic	39	60
Glottic	24	37
Subglottic	2	3
TNM Staging		
II	6	9
III	49	75.5
IV	10	15.5
Treatment approach		
Surgery	1	2
Radiotherapy	9	14
B/CTRT	49	75
ICT-B/CTRT	6	9

PS: Performance status-ECOG; B/CTRT: bio/chemoradiotherapy; ICT-B/CTRT: induction chemotherapy followed by bio/chemoradiotherapy.

**Table 2:** Laryngoesophageal dysfunction-free survival (LDS) and overall survival (OS) multivariate analysis of laryngeal cancer patients treated with preservation approaches

	LDS			OS		
Factors	HR	p-value	CI	HR	p-value	CI
Ki67	1.001	0.938	0.976-1.027	1.053	0.050	1.000-1.108
P53	0.982	0.209	0.956-1.010	1.016	0.477	0.972-1.062
SGLT	1.007	0.238	0.995-1.019	0.993	0.497	0.972-1.014
MAP17	32.66	0.001	4.352-245.1	21.73	0.010	2.071-228.1
PS=2	13.99	0.019	1.534-127.7	4.162	0.446	0.106-162.8
Pret. Tracheo.	1.476	0.431	0.560-3.890	1.849	0.505	0.304-11.26
RT	0.161	0.258	0.007-3.804	0.042	0.161	0.000-3.549
Bio/CTRT	0.046	0.080	0.001-1.452	0.109	0.447	0.000-33.00
ICT-bio/CTRT	0.047	0.101	0.001-1.805	0.014	0.169	0.000-6.054
Stage II	0.224	0.202	0.023-2.228	0.402	0.703	0.004-43.73
Stage III	0.636	0.500	0.171-2.367	0.048	0.016	0.004-0.569

Pret. Tracheo.: pretreatment tracheotomy required. PS: ECOG-performance status. B/CTRT: bio/chemoradiotherapy; ICT-B/CTRT: induction chemotherapy followed by bio/chemoradiotherapy.

### SGLT1 overexpression in human larynx tumors correlates with MAP17 levels

**Figure 3:**
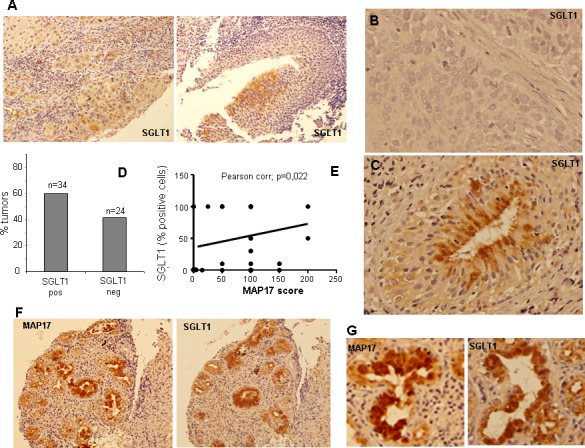
SGLT1 overexpression in larynx tumors A) Representative images are shown of SGLT1 immunostaining of different larynx tumors. B) High magnification of SGLT1 positive and C) negative tumors. D) Graph representing the percentage of larynx tumors positive or negative for SGLT1 expression. E) Graph representing the correlation between MAP17 and SGLT1 expression in each tumor. The statistical analysis was performed by Pearson correlation (p=0,0022). F) Samples from one patient showing clear correlation between the expression of MAP17 and SGLT1. G) High magnification of samples from one patient showing clear correlation between the expression of MAP17 and SGLT1.

Previous results indicate that MAP17-dependent tumorigenic properties depend on the indirect activation of ROS by SGLT1 transport and that there is a correlation between the expressions of both markers in cervix tumors [30]. Therefore, we measured SGLT1 expression levels in the same cohort of larynx tumor samples. We found that some tumors showed positive SLGT1 staining, with approximately 40% tumors being positive for SLGT1 (Figure 3A, B, C and D). However, only a few samples showed very high staining levels. The distribution of the SGLT1-positive tumors among the different larynx tumors showed a clear correlation with MAP17 expression (Figure 3E, F and G). Pearson indicator expressed a positive significant correlation between MAP17 and SGLT (P=0.3, p=0.022).

### MAP17 as predictive biomarker for laryngeal cancer

The high MAP17 group correlated in this study with better OS, LDS and locoregional control. When MAP17 was measured as a constant variable, multivariate Cox model demonstrated that higher rates of MAP17 levels correlated with improved OS (HR: 0.98, p= 0.001). Nevertheless, this could not be confirmed for LDS (HR 0.99, p=0.8), probably due to the limited number of cases.

**Figure 4:**
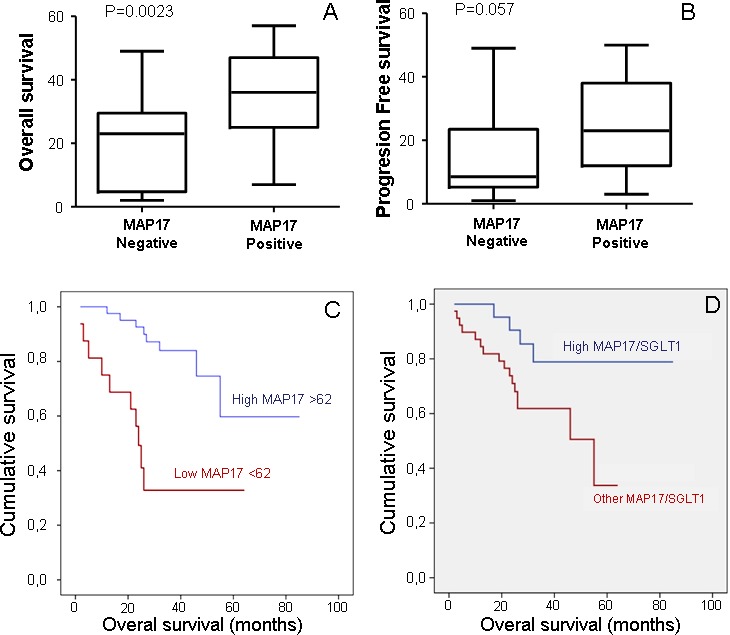
MAP17 alone or in combination with SGLT1 are good independent markers for patient survival A) Correlation of MAP17expression measured as a dichotomous variable, MAP17 high rates (>62) with overall survival. B) Correlation of MAP17expression measured as a dichotomous variable, MAP17 high rates (>62) with laryngoesophageal dysfunction-free survival. C) A Kaplan-Meier curve is shown indicating that MAP17 could be a good prognostic marker for overall survival in laryngeal tumor patients treated with radiotherapy plus bio/chemotherapy. D) A Kaplan-Meier curve shown indicates that combined high levels of MAP17 and SGLT1 levels are a good prognostic marker for survival in cervical tumor patients treated with radiotherapy plus adjuvant chemotherapy.

In order to distinguish a cutoff point for MAP17 levels a ROC curve was performed and punctuation of 62 score chosen. When measured as a dichotomous variable, MAP17 high rates (>62) were related with increased OS, LDS, and locoregional control. A difference of 35.3 months was observed between high MAP17 levels (67 months) and low MAP17 levels (31.7 m) in Kaplan-Meier model (IC 95%; p<0.001) and the HR estimator for high MAP17 was 0.78, p=0.002 in the multivariate analysis (Figure 4). Regarding LDS, high MAP17 showed a survival benefit of 13.1m (47.6 m vs. 34.5 m, p=0.002) with a HR: 0.14, p=0.003 in multivariate analysis. The effect of MAP17 high levels on the improved survival was significant after controlling for other variables: P53, Ki67, SGLT, PS, TNM, pretreatment tracheotomy and treatment received and shown in Table 2. Regarding locoregional control, patients with high MAP17 showed to have better outcomes than low MAP17 (53.9 m vs 44.5 m, p=0.016) and the results were confirmed in the multivariate model (p=0.045).

However, although MAP17 correlated with SGLT in the Pearson model (Figure 3E), SGLT alone did not show statistically significant correlation with OS or LDS (results not shown). Moreover, the associated high levels of MAP17 and SGLT did show improved OS than MAP17 alone (72.4 m vs 42m, p=0,028) (Figure 4D).

These data confirm that MAP17 alone, or preferably combined with SGLT1, is a good prognostic marker for survival in patients with larynx cancer treated with B/CTRT.

### Tumor cells overexpressing MAP17 are more sensitive to radiation

**Figure 5:**
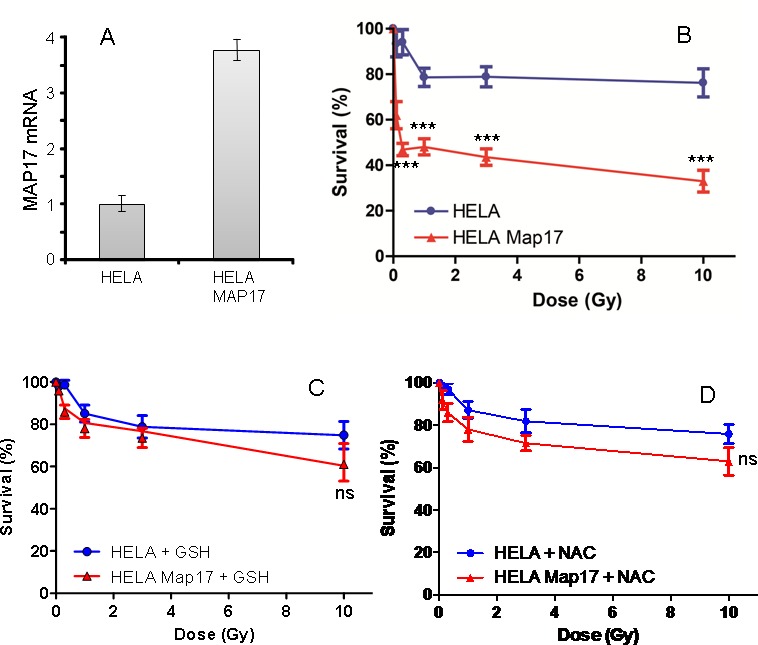
MAP17 overexpression in Hela cells induces sensitivity to radiotherapy A) Hela cancer cells expressing ectopic MAP17 cDNA were selected and analyzed for MAP17 mRNA expression by quantitative RT-PCR. B) Hela cells expressing ectopically MAP17 cDNA (Hela-Map17) and parental cells expressing only empty vector (Hela) were seeded at equal concentration and subjected to different radiation doses as indicated, in triplicate samples. 48 hrs after treatment the percentage of survival cells was measured in each case and plotted in the graph. The experiment was performed three independent times in triplicate. C and D) Hela cells expressing ectopically MAP17 cDNA (Hela-Map17) and parental cells expressing only empty vector (Hela) were seeded at equal concentration and subjected to pretreatment with 10 mM GSH (C) or 10 mM NAC (D) during 18 hrs, then treated with different radiation doses as indicated, in triplicate samples. 48 hrs after treatment the percentage of survival cells was measured in each case and plotted in the graph.

To explore whether MAP17 may be causal in this response, we expressed MAP17 cDNA in Hela cells and subjected these cells and their parental expressing only empty vector, to different doses of radiotherapy. Our data showed that Hela cells expressing MAP17 (Figure 5A) were more sensitive to radiation than parental cells without MAP17 (Figure 5B), therefore confirming the causal role of MAP17 in the sensitivity to radiation.

Finally, to test our initial hypothesis of the relevance of ROS in the MAP17-enhanced radiosensitivity of Hela cells, we treated the cells with antioxidants GSH and NAC, and subjected these cells to different radiation doses (Figure 5C and D). We observed that both antioxidant treatments reduced the sensitivity of MAP17-expressing Hela cells to a range similar to parental cells, which remains mostly unaltered (Figure 5C and D).

These data confirm the relevance of the oxidative status of the tumors in the response to radiation.

## DISCUSSION

MAP17 is a small 17 kDa non-glycosylated membrane protein overexpressed in human carcinomas [29]. Tumor cells with MAP17 overexpression show enhanced proliferative capabilities [31, 32]. MAP17 expression is associated with an SGLT-dependent ROS increase that acts as a second messenger enhancing tumorigenesis. While a mild increase in ROS has been shown to activate signaling cascades that upregulate tumorigenic processes, further ROS increases lead to a potentially toxic cellular environment and programmed cell death [33]. The hypothesis is that tumors expressing high levels of ROS producing MAP17 and SGLT1 proteins can benefit from therapies such as cisplatin or radiotherapy that increase oxidative stress and could sensitize them to cell death. In this setting, our analysis in laryngeal cancer showed a significant relationship between high MAP17 protein expression and increased OS, suggesting that MAP17 expression is an independent biomarker for survival. In fact, high MAP17 levels demonstrated better OS than low levels (67 months vs. 31.7 months, IC 95%; p<0.001). Our work also shows that patients with high MAP17 showed better locoregional control and LDS. Furthermore, the associated high levels of MAP17 and SGLT showed improved OS, better than MAP17 alone. These results are consistent with others presented in cervical cancer in which high levels of MAP17, better in combination with high SGLT1, correlated with improved patient survival after treatment [30]. Furthermore, proof of principle experiments *in vitro* demonstrated that antioxidant treatments reduced the sensitivity of MAP17-expressing Hela cells to a range similar to parental cells, confirming the relevance of the oxidative status of the tumors in the response to Radiation.

Our data confirm that MAP17 alone, and better in combination with SGLT1, is a good prognostic marker for survival in patients with larynx cancer treated with radiotherapy plus chemotherapy. Therefore, MAP17 could predict which patients may have better survival outcomes and would benefit from preservation approaches. Further prospective and controlled studies are needed in order to confirm our results and validate MAP17 as a novel biomarker of clinical use in larynx cancer.

## METHODS

### Patients characteristics and treatment

We evaluated 65 patients with larynx cancer and their treatment and evolution from August 2005 to February 2014. All patients expressed informed consent and the project was approved by the local ethical committee at HUVR. Patients received specific treatment in our institution while tumor samples were obtained from four different national hospitals where the diagnosis was made. Eligibility criteria for preservation approaches in this study include patients with stage II-IV laryngeal tumors that had no contraindication for chemotherapy and/or radiotherapy, significant cartilage destruction, or more than 2 cm of tumor infiltration in the base of the tongue. TNM Staging System for the Larynx (7^th^ ed., 2010) was used for tumor classification. Patients were mainly male (94%) with squamous carcinoma and good general condition (PS 0-1: 95%). Tumors were more frequently localized in the supraglottic (60%) and 75.5% were stage III. Interestingly, 32% of patients required pretreatment tracheotomy. Most of the patients were candidates for organ preservation with B/CTRT (75%), RT (14%), or ICT-B/CTRT (9%). Preferred treatment concurrent to radiotherapy was cisplatin 100 mg per square meter on days 1, 22, and 43 of radiotherapy (74%) followed by weekly cisplatin 40 mg per square meter (11%) and monoclonal antibody cetuximab (11%). Carboplatin was chosen for 4% of patients. Population characteristics and treatments are detailed in Table 1.

### Tissue acquirement and preparation

Formalin-fixed, paraffin-embedded tissue sections from 65 laryngeal carcinomas were selected with the collaboration of the Andalusian Health Care Biological Resource Centre. Histological characterization of all samples was done by Hematoxylin and Eosin staining, followed by immunohistochemistry (IHQ) analysis of tissue microarrays (TMA).

### Immunohistochemistry

Three-micrometer slices were sectioned from the TMA block and applied to coated, immunochemistry slides (DAKO, Glostrup, Denmark). The slides were baked overnight in a 56°C oven, deparaffinized in xylene for 20 min, rehydrated through a graded ethanol series and washed with PBS. A heat-induced epitope retrieval step was performed by heating a slide in a solution of sodium citrate buffer pH 6.5 for 2 min in a conventional pressure cooker. After heating, the slides were incubated with proteinase K for 10 min and rinsed in cool running water for 5 min. Endogenous peroxide activity was quenched with 1.5% hydrogen peroxide (DAKO) in methanol for 10 minutes, and incubation with the primary antibodies anti-MAP17 (1:4) [29-33] and anti-SGLT1 (Abcam #14685) was performed for 40 min. After incubation, immunodetection was performed with the EnVision (DAKO, Glostrup, Denmark) visualization system using diaminobenzidinechromogen as the substrate, according to the manufacturer's instructions. Immunostaining was performed in a TechMate 500 automatic immunostaining device (DAKO) and measured through a double-blind visual assessment using microscopic observation according to the anatomopathological experience of pathologists. Sample scoring was performed by semiquantitative microscopic analysis, considering the number of stained cells and signal intensity. Both MAP17 and SGLT stainings were performed. In both cases we used the score obtained by the multiplication of the intensity levels (1, 2 or 3) by the percentage of positive cells. For MAP17, the threshold used is the score of 62, obtained by ROC curve as the most relevant to establish as dichotomous variable. For SGLT1, there was no staining at all in non tumoral surrounding tissue, and the positivity was considered when positive cells were observed in the tumor.

### Cell culture

Hela malignant cervical tumor cells were obtained from the European Collection of Cell Cultures (ECACC) human cell line repository and maintained in Dulbeccós modified Eaglés medium (Sigma) containing 10% fetal bovine serum (Sigma), penicillin, streptomycin and fungizone. MAP17 full-length cDNA was cloned into pBabepuro and mass culture generated by stable gene transfer in Hela cells. After selection with 2 μg/ML puromycin, mass cultures were used for the study. As a control, Hela cells were transfected with pBabepuro alone and selected.

### Radiation treatment of *in vitro* culture

Cells were irradiated using Costar 24 well cell culture plates (Corning Incorporated, NY USA). To simulate actual radiobiological experimental conditions, each well was ﬁlled with culture medium. The plate dimensions were 12.5 × 8.5 cm. The inner diameter of the well was 16 mm and the distance between the centers of two neighboring wells was 20 mm. Plates were positioned inside a water-equivalent device, specifically designed to fit the plate. This device measures 16 × 16 × 2 cm, and is placed inside the IBA BodyPhantom (IBA Dosimetry GbmH, Schwarzenbruck, Germany) at a depth of 6 cm. Simulation was performed using a Toshiba Aquilion CT scanner (Toshiba Corporation, Japan). CT images were exported to the treatment planning system Philips Pinnacle V9.2 (Philips Radiation Oncology Systems, Madison, WI). Five plans were designed to deliver uniform doses of 0.1 Gray (Gy), 0.3 Gy, 1 Gy, 3 Gy and 10 Gy using static beams of 24 x18 cm. To verify the dose within every well, we delineated 24 regions of interest (ROI) that had a diameter of 16 mm. The ROI was estimated at the bottom of the wells and 5mm upwards. The dose delivered to the cells was verified with the IBA Compass system (IBA Dosimetry GbmH, Schwarzenbruck, Germany). Differences between the prescribed dose and the dose received were within 3%. The irradiation was delivered using 6 megaelectronvolts (MV) photon beams from an Elekta Synergy Linac (Elekta Oncology System, Ltd, Crawley, UK) with a dose rate of 500 mu/min.

### Statistical analysis and definitions

Kaplan-Meier method was used for survival analysis, using Cox Proportional Hazards model to adjust for the explanatory variables, obtain the p-values and estimate the HR. Multivariate logistic regression was used to obtain odds ratio (OR) and confidence intervals (CI 95%). Pearson's correlation measured dependence between quantitative variables. A receiver operating characteristic (ROC) curve was performed to assess MAP17 cutoff point (three year overall survival), which we checked using the optimal Youden index-based point. In addition, a log-rank test compared the survival distributions between the high MAP17 levels and the low levels. Statistical calculations were performed using SPSS 15.0 software.

OS has been defined as the length of time from the date of diagnosis until date of the last medical record. Adopting Lefebvre Larynx Preservation Consensus Panel (2009) for laryngoesophageal dysfunction-free survival (LDS), we considered endpoint events: death, local relapse, total or partial laryngectomy, tracheotomy at two or more years, or the presence of a feeding tube at two or more years [34]. Locoregional control was considered from the date of the diagnosis until local progression or last medical record, after excluding patients who developed distant metastases or died due to other causes not related to the larynx tumor.
